# Influence of the cardiac glycoside digoxin on cardiac troponin I, acid–base and electrolyte balance, and haematobiochemical profiles in healthy donkeys (*Equus asinus*)

**DOI:** 10.1186/1746-6148-10-64

**Published:** 2014-03-12

**Authors:** Mohamed Tharwat, Fahd Al-Sobayil

**Affiliations:** 1 Department of Veterinary Medicine, College of Agriculture and Veterinary Medicine, Qassim University, Qassim, Saudi Arabia; 2 Permanent address: Department of Animal Medicine, Faculty of Veterinary Medicine, Zagazig University, Zagazig, Egypt

**Keywords:** Cardiac troponin I, Digoxin, Donkey, Heart failure, Intoxication

## Abstract

**Background:**

The effect of digoxin administration on the serum concentration of the cardiac troponin I (cTnI) has not been reported to date in equidae. This study was therefore designed to evaluate the effect of digoxin on cardiac cell damage in donkeys (*Equus asinus*) as assessed by cTnI, acid–base and electrolyte balance and haematobiochemical profiles. Ten clinically healthy donkeys were given an IV infusion of digoxin at a dose of 14 μg/kg. Blood samples were collected from the donkeys up through 72 h post-injection.

**Results:**

Three of the donkeys exhibited increased heart and respiratory rates post-injection. In the other seven animals, the heart and respiratory rates were lower 4 h post-injection. The serum digoxin concentration increased significantly at many time points after injection. The serum concentration of cTnI did not differ significantly between pre- and post-injection. An increase in blood pH was noted at 3 h after digoxin injection. There were also increases in PO_2_ and in oxygen saturation. Decreases in PCO_2_ at 2 to 48 h post-injection as well as a decrease in blood lactate at 4 h post-injection were observed. The serum concentration of glucose remained significantly elevated at all-time points after digoxin injection.

**Conclusions:**

It is concluded that administration of digoxin to healthy donkeys (14 μg/kg) did not result in elevations of serum cTnI concentration, signs of digoxin intoxication, ECG abnormalities and did not increase serum concentrations of blood urea nitrogen and creatinine.

## Background

Digoxin is the most commonly used cardiac glycoside [1]. It is a well-tolerated and inexpensive drug which has been in wide use for many years as a treatment for heart failure and arrhythmia. Although a number of sophisticated management options and new therapeutic agents have been developed in recent years for the treatment of patients with heart failure, digoxin is still one of the most frequently prescribed drugs and remains the first choice of treatment [2-4].

In equidae, digoxin is used in the treatment of naturally-acquired heart failure [5]. Treatment with digoxin results in an increase in cardiac contractility and a decrease in heart rate, with increased myocardial oxygen consumption, increased cardiac output and decreased cardiac size [6]. The improvement in cardiac output promotes diuresis and the reduction and elimination of oedema. The half-life of digoxin in the horse is 17–23 h [7]. The proposed therapeutic, nontoxic range of digoxin in horses is calculated as follows: IV loading 14, IV maintenance 7, oral loading 70, and oral maintenance 35 μg/kg/24 h [7].

Cardiac troponins, especially cardiac troponin I (cTnI), are highly sensitive and specific markers of myocardial injury in veterinary medicine [8-11]. In horses, cTnI is elevated in animals undergoing training and endurance exercise [12-14], horses with cardiac diseases [15], horses with muscular disease [16], and in horses with monensin intoxication [11]. Measurement of cTnI concentrations has also been used recently for prognostication in horses undergoing emergency abdominal surgery [17].

The effect of digoxin administration on the serum concentration of cTnI has not been reported to date in equidae. The present study was therefore designed to investigate the influence of the cardiac glycoside digoxin on cardiac cell damage as assessed by cTnI, using donkeys (*Equus asinus*) as an equine model. The second objective was to investigate the effects of digoxin injection on the acid–base and electrolyte balance and on the haematobiochemical profiles in donkeys.

## Methods

### Animals, history and physical examination

The experimental protocol and all procedures used in this study were approved by the Ethics Committee for Animal Research of the Scientific Research Deanship of Qassim University, Saudi Arabia. In addition, animals were treated according to the regulations of the *Laboratory Animal Control Guidelines* of Qassim University, which basically conform to the *Guide for the Care and Use of Laboratory Animals* of the National Institutes of Health in the USA (NIH publications No. 86 to 23, revised 1996). Ten male donkeys (age 7.6 ± 2.4 y; weight 116 ± 17 kg) were used. The animals were selected on the basis of absence of any disease. Each donkey had received a full clinical examination, with special attention to the cardiovascular system. Animals were considered healthy based on physical examination, laboratory evaluation (normal complete blood cell counts and biochemistry panel), echocardiography and electrocardiography (ECG).

### Administration of digoxin and blood sampling

Digoxin^a^ was administered as an IV infusion at 14 μg/kg, a dose recommended for horses with heart failure [7,18,19]. Throughout the experiment, eight blood samples (T0-T7) were obtained from each animal. The first blood sample (T0) was collected immediately prior to injection of digoxin. Four blood samples were collected at 1 h (T1), 2 h (T2), 3 h (T3) and 4 h (T4) post-injection. Three additional blood samples (T5-T7) were obtained at the following 24, 48 and 72 h. At each sampling time, 10 mL of jugular blood was collected using sterile vacutainers. A 2-mL volume of blood was collected into a heparinised tube for determining blood gas parameters, 2-mL in EDTA tubes for haematological analyses, and the remaining 6 mL of blood was placed in plain tubes to obtain serum for the determination of digoxin, cTnI concentrations and other biochemical analytes.

### Electrocardiogram recording

A lead II electrocardiograph^b^ with a paper speed of 25 mm/s and a sensitivity of 10 mm/mV (1 cm = 1 mV) was used to constantly monitor the donkeys for the presence of arrhythmias during the entire study period. Alligator clips fixed to the electrocardiographic leads were attached directly to the skin after vigorous application of an electrode paste. The limb lead placement consisted of four electrodes: two just above the point of the elbow (right and left) and two on the stifle (right and left). The forelimbs were kept parallel to each other and perpendicular to the long axis of the body. The donkeys were kept standing and insulated from the ground by means of a rubber mat.

### Blood gas analyses

The heparinised blood samples were used immediately to analyse the acid–base and blood gas parameter values *in situ* using a portable clinical veterinary analyser.^c^ In this way, blood pH, partial pressure of carbon dioxide (PCO_2_), oxygen partial pressure (PO_2_), bicarbonate (HCO_3_^-^), anion gap base excess (BE), oxygen saturation (SO_2_), sodium, potassium, chloride and lactate were determined immediately in order to prevent changes in the concentrations of these parameters [20].

### Haematology and serum biochemistry

Haematological examinations [total and differential leukocyte count, red blood cell count (RBCs), haemoglobin, haematocrit, mean corpuscular volume (MCV), mean corpuscular haemoglobin (MCH), mean corpuscular haemoglobin concentration (MCHC) and platelet count] were carried out using an automated analyser.^d^ The serum samples were used to determine the concentrations of total protein, albumin, globulin, blood urea nitrogen (BUN), calcium, glucose, creatinine and total bilirubin. The serum activity of γ-glutamyl transferase (GGT), aspartate aminotransferase (AST), and creatine kinase (CK) was also measured. An automated biochemical analyser was used for the measurement of the biochemical parameters^e^.

### Cardiac troponin I and digoxin assays

Cardiac troponin I was analysed in serum using a point-of-care analyser according to the manufacturer’s instructions.^c^ This analyser employs a two-site enzyme-linked immunosorbant assay. All results are expressed as nanograms per millilitre (ng/mL) with an intra-assay coefficient of variance of 5%. The lower limit of detection of cTnI for this assay is 0.02 ng/mL. The serum concentrations of digoxin were determined using an electrochemiluminescent immunoassay-kit^f^ with a measuring range of 0.15 - 5.0 ng/mL. The intra- and inter-assay coefficients of variance for digoxin were 5.2 and 7.7%, respectively. The coefficients of variance for cTnI and digoxin assays were developed in human samples.

### Statistical analysis

Data from the 10 donkeys are presented as means ± standard deviation, and comparisons among T0-T7 values were conducted using SPSS program software [21]. The normality of the data was tested by the Kolmogorov–Smirnov test. Data were analysed using repeated measures analysis, with Fisher’s protected least significant difference (LSD) as the post-ANOVA test. The level of significance was tested at *P* < 0.05.

## Results

**Figure 1:**
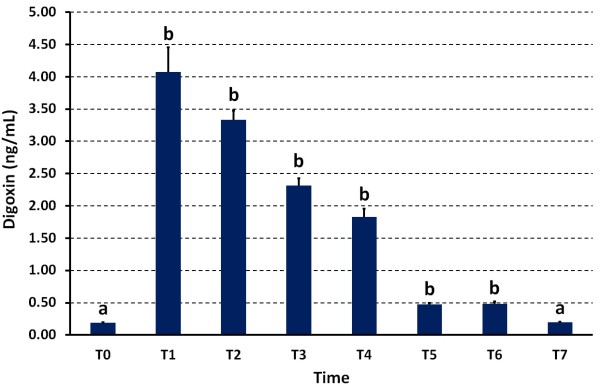
**Mean (±standard deviation) concentrations of serum digoxin in donkeys (*n*** **= 10) before injection of digoxin (T0) and at 1, 2, 3, 4, 24, 48 and 72 h post injection (T1-T7). **^a,b^Differ significantly.

None of the donkeys showed clinical abnormalities following injection of digoxin. The rectal temperature and heart and respiratory rates did not differ significantly after digoxin administration. In addition, the ECG results showed no cardiac arrhythmias in any of the donkeys by 72 h post-injection of digoxin. Figure 1 shows the serum concentration of digoxin in the donkeys before and after digoxin infusion. Serum digoxin concentration significantly increased at many time points after digoxin had been administered intravenously. At 1 h (T1), the digoxin concentration had increased to 4.1 ± 0.12 compared to 0.19 ± 0.02 ng/mL pre-injection (*P* < 0.0001). From the time points T2 to T6, gradual decreases of digoxin concentrations were observed until they returned to pre-injection values by T7. At all-time points (T0-T7), the serum concentrations of cTnI in the donkeys before and after digoxin infusion was ‘below detection limit’ for the assay and below established normal values using this assay.

Table 1 summarises mean ± SD of acid–base and electrolyte parameters in the donkeys after injection of digoxin. At T3, there was an increase in blood pH (*P* < 0.05). At T2-T5, there were decreases in PCO_2_, while at T3-T6, there were increases in PO_2_ (*P* < 0.05). Oxygen saturation increased at T3-T6 (*P* < 0.01). At T1-T4, there were decreases in blood lactate (*P* < 0.05). Other blood gas parameters including sodium, potassium, chloride, BE, HCO_3_^-^ and anion gap did not differ significantly compared to the pre-injection values (*P* > 0.05).

**Table 1:** **Mean values (± standard deviation) of acid–base and electrolyte parameters in donkeys (*n*** **= 10) injected with digoxin**

**Time**	**pH**	**PCO**_**2 **_**(mmHg)**	**PO**_**2 **_**(mmHg)**	**BE (mmol/L)**	**HCO**_**3**_**¯ (mmol/L)**	**SO**_**2 **_**(%)**	**Sodium (mmol/L)**	**Potassium (mmol/L)**	**Chloride (mmol/L)**	**AG (mmol/L)**	**Lactate (mmol/L)**
T0	7.42 ± 0.02	42.0 ± 2.6	31.2 ± 1.9	2.6 ± 1.0	27.1 ± 0.9	60.5 ± 3.4	132 ± 3	4.5 ± 0.3	104 ± 2.9	9.3 ± 0.4	1.9 ± 0.5
T1	7.43 ± 0.03	41.7 ± 3.3	30.7 ± 2.0	3.2 ± 1.5	27.6 ± 1.2	56.0 ± 7.3	133 ± 4	4.6 ± 0.5	105 ± 3.8	10.3 ± 1.6	1.0 ± 0.2^b^
T2	7.45 ± 0.04	39.4 ± 2.8^b^	30.0 ± 0.8	2.9 ± 1.9	27.1 ± 1.4	60.5 ± 1.2	132 ± 5	4.4 ± 0.5	105 ± 3.4	8.6 ± 0.9	1.2 ± 0.4^b^
T3	7.45 ± 0.01^b^	38.3 ± 0.8^b^	33.6 ± 2.1^b^	2.9 ± 2.4	26.8 ± 1.7	68.5 ± 3.2^b^	132 ± 3	4.8 ± 0.6	105 ± 3.7	8.8 ± 1.1	1.4 ± 0.6^b^
T4	7.44 ± 0.02	37.0 ± 2.2^b^	33.8 ± 1.2^b^	2.0 ± 1.5	26.2 ± 1.4	65.7 ± 4.6^b^	131 ± 3	4.2 ± 0.5	106 ± 3.5	9.5 ± 2.2	1.2 ± 0.3^b^
T5	7.42 ± 0.01	39.7 ± 2.3^b^	45.7 ± 16.6^b^	2.4 ± 1.3	26.6 ± 1.4	74.4 ± 10.9^b^	133 ± 4	5.0 ± 1.0	103 ± 2.8	8.7 ± 0.4	1.7 ± 0.6
T6	7.42 ± 0.03	40.6 ± 3.7	39.4 ± 11.4^b^	2.0 ± 0.6	26.4 ± 1.0	70.9 ± 10.2^b^	132 ± 3	4.9 ± 0.6	105 ± 3.4	9.1 ± 1.2	1.8 ± 0.4
T7	7.43 ± 0.02	41.8 ± 1.9	31.4 ± 3.7	3.4 ± 2.4	28.0 ± 2.2	62.7 ± 9.0	134 ± 3	5.1 ± 0.7	104 ± 3.9	9.3 ± 0.9	1.9 ± 0.2

PCO_2_, partial pressure of carbon dioxide; PO_2_, partial pressure of oxygen; BE, base excess; HCO_3_¯, bicarbonate; SO_2_, oxygen saturation; AG, anion gap. T0, immediately prior to injection of digoxin; T1-T7, 1, 2, 3, 4, 24, 48 and 72 h after digoxin administration. ^b^Differ significantly at *P* < 0.05 compared to pre-injection values.

Compared to pre-injection values, none of the haematological parameters showed a statistically significant difference after digoxin injection (Table 2). Table 3 shows the biochemical parameters in the donkeys before and for the 72 h post-injection of digoxin. The serum concentration of glucose remained significantly elevated (*P* < 0.05) at all-time points (T1-T7) after digoxin infusion. Other biochemical parameters did not differ significantly among all time points.

**Table 2:** **Mean values (± standard deviation) of haematological variables in donkeys (*n*** **= 10) injected with digoxin**

**Time**	**WBC (×10**^**9**^**/L)**	**Lym (×10**^**9**^**/L)**	**Mon (×10**^**9**^**/L)**	**Neu (×10**^**9**^**/L)**	**Eos (×10**^**9**^**/L)**	**Bas (×10**^**9**^**/L)**	**RBC (×10**^**12**^**/L)**	**HGB (g/dL)**	**HCT (%)**	**MCV (fl)**	**MCH (pg)**	**MCHC (g/dL)**
**T0**	15.5 ± 4.1	4.6 ± 1.6	0.7 ± 0.4	8.2 ± 1.6	1.7 ± 1.1	0.2 ± 0.1	7.6 ± 0.3	13.3 ± 1.0	39.3 ± 3.7	51.4 ± 3.7	17.4 ± 1.1	33.9 ± 1.3
**T1**	16.1 ± 6.0	3.8 ± 1.7	1.0 ± 0.6	9.2 ± 3.3	1.9 ± 1.3	0.1 ± 0.1	7.4 ± 0.6	12.5 ± 1.3	37.2 ± 4.2	50.5 ± 5.0	17.0 ± 1.8	33.7 ± 1.2
**T2**	14.1 ± 4.0	3.5 ± 1.3	0.6 ± 0.3	8.4 ± 1.8	1.7 ± 1.2	0.2 ± 0.1	7.2 ± 0.8	12.1 ± 2.3	35.8 ± 3.9	50.7 ± 4.7	17.2 ± 1.8	33.8 ± 1.2
**T3**	13.3 ± 5.7	3.4 ± 1.8	0.5 ± 0.3	7.8 ± 3.0	1.7 ± 1.3	0.2 ± 0.1	6.6 ± 1.4	11.1 ± 2.2	33.5 ± 5.6	51.0 ± 4.7	17.0 ± 1.6	33.2 ± 0.9
**T4**	16.0 ± 4.4	3.0 ± 2.3	0.8 ± 0.4	10.6 ± 3.4	1.5 ± 1.2	0.2 ± 0.1	6.9 ± 1.9	11.7 ± 2.3	34.8 ± 5.1	51.0 ± 4.5	17.2 ± 1.5	33.7 ± 0.4
**T5**	16.6 ± 4.0	4.4 ± 2.1	0.7 ± 0.3	10.2 ± 3.5	1.4 ± 1.3	0.2 ± 0.1	7.3 ± 0.6	12.7 ± 1.3	37.1 ± 4.9	50.0 ± 5.1	17.3 ± 1.3	34.5 ± 1.0
**T6**	15.7 ± 5.3	3.4 ± 1.0	0.8 ± 0.6	9.8 ± 3.1	1.5 ± 1.2	0.2 ± 0.1	7.2 ± 0.6	12.4 ± 1.2	36.2 ± 3.8	50.0 ± 4.1	17.2 ± 1.3	34.4 ± 0.6
**T7**	15.9 ± 4.4	4.6 ± 1.9	0.6 ± 0.2	9.1 ± 1.9	1.7 ± 1.2	0.2 ± 0.1	7.3 ± 0.7	12.6 ± 1.2	36.5 ± 4.6	50.0 ± 4.3	17.2 ± 1.2	34.6 ± 0.9

WBC, white blood cells; Lym, lymphocyte; Mon, monocyte; Neu; neutrophil; Eos, eosinophil; Bas, Basophil; RBC, red blood cells; HGB, hemoglobin; HCT, hematocrit; MCV, mean corpuscular volume; MCH, mean corpuscular hemoglobin; MCHC, mean corpuscular hemoglobin concentration; PLT, platelets. T0, immediately prior to injection of digoxin; T1-T7, 1, 2, 3, 4, 24, 48 and 72 h after digoxin administration.

**Table 3:** **Mean values (± standard deviation) of biochemical variables in donkeys (*n*** **= 10) injected with digoxin**

**Time**	**Glucose (mmol/L)**	**CA (mmol/L)**	**BUN (mmol/L)**	**CRE (μmol/L)**	**AST (U/L)**	**CK (U/L)**	**GGT (U/L)**	**TBIL (μmol/L)**	**TP (g/L)**	**ALB (g/L)**	**GLOB (g/L)**
**T0**	3.8 ± 0.6	3.0 ± 0.1	6.1 ± 0.6	44 ± 8	618 ± 157	1339 ± 1275	56 ± 10	2.2 ± 0.3	73.5 ± 7.2	35.9 ± 4.4	37.6 ± 11.1
**T1**	4.6 ± 0.9^b^	2.9 ± 0.1	6.1 ± 0.4	44 ± 9	632 ± 137	1356 ± 1280	58 ± 14	2.5 ± 0.4	75.0 ± 5.6	45.4 ± 14.2	36.2 ± 11.2
**T2**	5.1 ± 0.8^b^	3.0 ± 0.1	6.2 ± 0.4	42 ± 12	627 ± 136	1326 ± 1204	55 ± 8	2.5 ± 0.4	74.4 ± 4.3	37.9 ± 5.8	36.6 ± 9.7
**T3**	5.2 ± 0.8^b^	3.0 ± 0.2	6.2 ± 0.5	44 ± 13	644 ± 114	1219 ± 1117	49 ± 12	2.8 ± 0.8	74.0 ± 3.7	37.8 ± 5.9	36.1 ± 9.0
**T4**	6.2 ± 1.1^b^	2.9 ± 0.3	6.2 ± 0.4	43 ± 13	791 ± 226	1232 ± 1102	52 ± 11	2.5 ± 0.4	74.6 ± 4.6	36.9 ± 4.2	38.1 ± 8.0
**T5**	5.3 ± 0.9^b^	3.1 ± 0.2	6.0 ± 0.7	37 ± 3	778 ± 243	2335 ± 2402	50 ± 9	2.2 ± 0.3	77.2 ± 5.1	39.6 ± 4.6	38.1 ± 9.0
**T6**	5.8 ± 1.2^b^	3.0 ± 0.1	6.1 ± 0.4	39 ± 9	743 ± 240	1560 ± 980	51 ± 8	2.5 ± 0.4	75.2 ± 6.4	37.5 ± 5.7	37.3 ± 11.3
**T7**	4.9 ± 0.5^b^	3.0 ± 0.1	6.4 ± 0.5	44 ± 8	576 ± 186	1072 ± 713	60 ± 13	2.5 ± 0.7	74.6 ± 7.0	39.5 ± 5.8	34.9 ± 12.1

CA, calcium; BUN, blood urea nitrogen; CRE, creatinine; AST, aspartate aminotransferase; GGT, γ-glutamyl transferase; CK, creatine kinase; TBIL, total bilirubin, TP, total protein; ALB, albumin; GLOB, globulin. T0, immediately prior to injection of digoxin; T1-T7, 1, 2, 3, 4, 24, 48 and 72 h after digoxin administration. ^b^Differ significantly at *P* < 0.05 compared to pre-injection values.

## Discussion

To the authors’ knowledge, this is the first study to evaluate the effect of digoxin on cardiac cell damage as assessed by cTnI, acid–base and electrolyte balance and the haematobiochemical profiles in donkeys (Equus asinus) as an equine model. Before injecting digoxin, the mean serum cTnI in the donkeys was 0.004 ng/mL, a value ‘below detection limit’ of the assay and below established normal values in healthy horses (0.06-0.1 ng/mL) using this assay [11,12,14,22]. However, we don’t know for sure whether we can apply the normal values of horses for donkeys. At no time point after injecting digoxin was cTnI significantly different compared to pre-injection values (P > 0.05).

Cardiac troponin I is a protein found in the myocardial cells. Its serum concentration elevates after acute myocardial injury because of leakage from the damaged myocardial cells [23,24]. It is the ‘gold standard’ for the non-invasive diagnosis of myocardial injury in humans [25,26]. Similarly, in animals cTnI has a high sensitivity and specificity in patients with primary or secondary cardiac disease [8,24]. Persistently increased cTnI blood concentration suggests ongoing active and irreversible damage to cardiomyocytes [8,24]. The degree of increase has been shown to be correlated with the extent of myocardial damage and with survival in human [27] and animal [28].

In humans, the therapeutic and toxic plasma concentrations of digoxin are usually set at 0.8 - 1.6 ng/mL and greater than 2.4 ng/mL, respectively [29]. Similar numbers have been derived for animals: e.g., digoxin plasma concentrations of 0.5 - 2.0 ng/mL are nontoxic in horses [7] and a 2.3 ng/mL concentration is nontoxic in cats [30]. In dogs, plasma digoxin concentrations up to 2.5 ng/mL are nontoxic, but concentrations of 2.5 - 3.0 ng/mL are associated with increased probability of toxicosis [31]. In donkeys, however, the therapeutic and toxic plasma concentrations of digoxin have not been reported in the literature.

Digitalis intoxication is characterised by several clinical signs varying from mild gastrointestinal upset (anorexia, vomiting, and diarrhoea) to chronic weight loss and life-threatening arrhythmias [32,33]. Increased serum BUN and creatinine are interpreted as evidence that digoxin toxicity has compromised renal function. In this study, serum digoxin concentration had increased in the donkeys at T1 to 4.08 ± 0.12 ng/mL compared to 0.19 ± 0.02 ng/mL pre-injection. Thereafter, digoxin concentration decreased steadily to reach 1.83 ± 0.40 ng/mL 4 h post-injection. During that time and up to 72 h post-injection, none of the donkeys showed signs of digoxin intoxication, cardiac arrhythmias, ECG abnormalities or elevated serum BUN and creatinine. Therefore, it can be assumed that the toxic serum digoxin concentration in donkeys would be higher than in humans, horses, dogs and cats. Thus, another study is needed to verify the toxic serum digoxin concentration in donkeys.

The metabolic alkalosis observed 3 h after digoxin injection may have been due to decreases in PCO_2_ and lactate. The decreased PCO_2_ and the increased PO_2_ and SO_2_ after injection may have resulted from the increased respiratory rates. Lactate is known as the end product of anaerobic glycolysis, a pathway that is of key importance during normal metabolic and athletic events. Aside being a waste product during high intensity exercise, lactate is also a valuable substrate that significantly contributes to the energy production of heart, liver, kidneys, non-contracting muscles and even brain [34]. In the present study, lactate concentration decreased during the 4 h (T1-T4) post-injection of digoxin, and this may have been caused by the decrease in the metabolic index due to the inactivity of the animals during these time points. Similarly, in healthy humans, lactate concentration was reported to be stable following digoxin infusion [35]. Concerning the biochemical variables, hyperglycaemia was found in the donkeys after digoxin administration. In human medicine, it is well-known that digoxin therapy increases the adrenaline-induced, and attenuates the insulin-induced hypoglycaemia due to catecholamines release in the acute stage of the action of digitalis glycosides [36].

This study has its own limitations. One of these limitations is the small sample size; therefore the results of this study are preliminary and should be interpreted with caution. Another limitation of this study is the using of healthy rather than diseased donkeys. Braunwald [37] had reported that there is a direct correlation between the level of myocardial impairment and the effectiveness of digoxin therapy on cardiac function. Although the i- STAT^®^ cTnI assay was initially designed for use in humans, there is good reason to anticipate cross reactivity based on the highly conserved nature of the cardiac troponins across mammalian species [38-40]. While it may not be practical in veterinary medicine to develop species-specific assays for all relevant species, this practical assumption should come with an acknowledgement of less than perfect validation of this assay. In this study, we thought that the i- STAT^®^ cTnI assay would detect the donkeys’ cTnI, because, despite the lack of a known donkey cTnI amino-acid sequence, a third limitation of this study, the sequence of cTnI is highly conserved among all species. In addition, the i- STAT^®^ analyser has been clinically validated against reference analysers in other species [11,41,42]. In addition, the high homology of amino-acid sequences between mammals within the sequence regions detected by cTnI analysers allows veterinarians to measure cTnI concentrations in many animal species with the same equipment as has been developed for humans, rather than necessitating the development of species-specific technology. As all the troponin samples in this study were below the detection level of the test, therefore the assay may not have had sufficient sensitively to detect small changes in cTnI levels below the detection level. These small elevations may still be of clinical relevance.

## Conclusions

Although the serum digoxin concentrations had increased in the donkeys to 4.08 ± 0.12 ng/mL 1 h after injection, the donkeys showed no sign of digoxin intoxication, ECG abnormalities or elevated serum BUN and creatinine. It is therefore assumed that in donkeys, the toxic serum digoxin concentration is higher than in humans, horses, dogs and cats [7,29-33]. Consequently, another study is warranted to verify the toxic serum digoxin concentration in healthy donkeys and in those with cardiac diseases.

## Endnotes

^a^Lanoxin, GlaxoSmithKline S.P.A, Parma, Italy.

^b^Kenz Cardico 302, Suzuken Co., Ltd., Japan.

^c^VetScan i-STAT 1, Abaxis, California, USA.

^d^VetScan HM5, Abaxis, California, USA.

^e^VetScan VS2, Abaxis, California, USA.

^f^Roche Diagnostics, Indianapolis, Indiana, USA.

## Competing interests

The authors declare that they have no competing interests.

## Authors’ contributions

MT initiated and planned the study. MT and FA carried out the experimental and laboratory work. MT wrote the manuscript and made the figure and tables and revised the manuscript. FA read and revised and approved the manuscript. Both authors read and approved the final manuscript.
